# Hospitals and Their Special Needs

**Published:** 1918-06-22

**Authors:** 


### HOSPITALS AND THEIR SPECIAL NEEDS.

**Brentwood District Cottage Hospital** is a local institution, entirely supported by voluntary contributions. There are thirteen beds and a resident staff of four nurses. The cost of running the hospital is about £800 a year, and funds are badly needed.

**British Hospital for Mothers and Babies, and National Training School for District Midwives, Woolwich.**—Only waiting for the end of the war to build. Three-acre freehold site secured. Building fund, £20,000; £10,000 still needed. Training specially adapted to the needs of district midwives. Minimum course to candidates without general training, one year.

**Chelsea Hospital for Women, Fulham Road, S.W.**—The debt on the new hospital at present stands at £11,000. The Council trust that by the generous help of many who are becoming acquainted with the good work of the institution this sum may be reduced in the current year.

**City of London Hospital for Diseases of the Chest, Victoria Park, E. 2.**—This hospital is called upon to meet still greater demands. Part of its accommodation is reserved for the treatment of heart and other non-tuberculous chest complaints, but the increased ravages of consumption which unfortunately have to be faced cause a great pressure upon the beds. In providing for the many cases of ex-soldiers for whom admission is sought, the committee is doing its utmost to avoid interference with its ordinary functions. A division between the civil and the military needs in this respect can hardly be made under present conditions, but the committee rightly lay stress on the importance of not reducing the facilities for treatment of the civil population, which includes the wives and children of our sailors and soldiers. The expenditure is increased by over £100 per week, and it is feared that without much increased support the present year will show a serious adverse balance.

**City of London Lying-in Hospital, City Road, E.C.**—The work of the City of London Lying-in Hospital during the past year was carried on in the face of great difficulties, but in spite of these the number of patients treated greatly exceeded the record for any other year of the hospital's history. This fact furnishes positive proof that the hospital's work is becoming increasingly important, and that it is appreciated more than ever by the poor people to whom its benefits are extended. The financial position of the hospital is now very serious, for there was a large deficit at the end of last year, and, in addition, the loan from the bankers now stands at £7,300, the yearly interest on which amounts roughly to £350.

**East End Mothers' Lying-in Home, Commercial Road, E.**—

"We take them in this Home of Life, and where
They go, the Angels bend to bless
The mother treasures left by brave men in our care.
Friends, give us help, that we may do our share
To tend the mothers in their sore distress,
Thus turn to sweetness half their bitterness."

**East Ham Hospital, E. 7.**—The hospital was opened in 1902, is supported entirely by voluntary contributions, and deals mainly with accident cases. During the war ten beds are reserved for wounded soldiers. The special need just now is money to complete the extension of the grounds of the hospital.

**East London Hospital for Children, Shadwell, E. 1**—In spite of increased financial support, expenses have risen so enormously that the hospital is practically no better off. The lives of children are so very important at this present juncture that every effort should be made by the State to secure adequate support for children's hospitals.

**Eltham and Mottingham Cottage Hospital.**—The hospital stands in good repute in the neighbourhood, which since the war has by reason of its proximity to Woolwich largely increased, hundreds, indeed thousands of people having been added to the population. This has naturally increased the number of patients, and has also, though not in an equivalent degree, increased the subscribers of small amounts, but unfortunately many subscribers of more substantial sums have left the neighbourhood. It is claimed that whilst the patients have every comfort and good food in plenty, the hospital is run with great economy.

**Epsom and Ewell Cottage Hospital, Surrey.**—During the last four years the expenses of this hospital have exceeded the ordinary income by £469. At the end of 1917 there was nearly £700 in the reserve fund, but £300 of this has since been spent, and it is feared that the fund will be further encroached upon before the end of the year.

**Great Northern Central Hospital,** with its auxiliary branches, contains 430 beds. Last year it treated 3,250 in-patients, and out-patient attendances numbered 104,667. Three thousand wounded soldiers have been treated in the wards since the outbreak of war. It is a general hospital, with special departments for specific diseases. The Earlsmead Home of Recovery, Highgate, and the Reckitt Convalescent Home, Clacton-on-Sea, are branches, with twenty-five and sixty beds respectively. It is estimated that £45,000 will be required this year, of which only £1,200 is obtainable from endowments.

**Grosvenor Hospital for Women, Vincent Square, Westminster.**—The needs of this hospital are many and its difficulties great. Subscriptions and donations towards maintenance are especially asked for.

**Guy's Hospital, Southwark, S.E.**—For the first time in the history of the institution the ordinary expenditure has exceeded £100,000 in the year, and the accounts show a deficiency of nearly £13,000. Every heading on the expenditure side shows an increase, but the high cost of provisions is the outstanding feature, and under present conditions it is not capable of much relief. As an illustration of the effect of war conditions, it may be stated that the cost for board of the female staff per head was in 1914 £16 3s. 7d., in 1917 £26 16s. 11d. At the present time public calls for generosity are on every hand, but it is undoubted that circumstances have brought to many a greater capacity for ministering to the needs of others. While the governors are full of gratitude to those who by their gifts for endowment are doing so much to make the future of the hospital secure, they hope that the present needs of the in-

**HOSPITALS AND THEIR SPECIAL NEEDS—**
(*Continued.*)

stitution may not escape recognition and the encumbrance of a debt may be avoided.

**Hampstead General and North-West London Hospital, Haverstock Hill, N.W. 3.**—The hospital maintains 137 beds, of which fifty-three are reserved for soldiers. The average weekly number of beds at present occupied is 121. The hospital is dependent entirely upon voluntary contributions for support; the income derived from invested funds amounts to £650 only.

**Hendon Cottage Hospital (King Edward Memorial).**—The cost per in-patient per week rose from £2 2s. 10d. in 1916 to £2 11s. in 1917. Enlargement is needed, but a comprehensive scheme cannot be proceeded with at present. It is proposed, however, to add verandahs to two wards, which will provide several extra beds.

**Hospital for Epilepsy and Paralysis, Maida Vale and Golders Green,** has 225 beds for the treatment of nervous diseases. Of these beds 140 at Golders Green are, by arrangement with the British Red Cross Society and the Ministry of Pensions, for the benefit of discharged soldiers suffering from shell shock, and thirty-five at Maida Vale are reserved for soldiers suffering from nervous disorders, specially labelled in France, and sent direct to the hospital. The charity needs close on £10,000 per annum to maintain this beneficent work.

**Hospital for Sick Children, Great Ormond Street, W.C.**—The fact that 202 of the 204 available beds are on the average occupied each day of the year speaks clearly for the usefulness of this hospital for little ones. The out-patient department is correspondingly busy. Never has the call to save the children been so important as it is to-day, and it is a call that cannot be neglected. It would be a tragedy if the hospital had to limit its operations for lack of support. The institution asks for help to "carry on" as successfully as it has done for three-quarters of a century.

**King's College Hospital, Denmark Hill, S.E. 5.**—An appeal for £100,000 to pay off the debt has been made, since which the final accounts have been received showing that a further £35,000 is needed to defray the total cost of the erection of the hospital. Up to the present time about £34,000 has been received in response to the appeal, thus leaving £101,000 to be raised. It is earnestly hoped that all friends of King's College Hospital will respond liberally to this appeal, so that the cost of the erection of the new hospital may be provided. In addition to the sum required for this purpose the ordinary income must be increased by at least £17,000 per annum that the work of the hospital may be efficiently carried on and the increased cost of general maintenance due to the ever-advancing price of all commodities duly met. Will you help? No contribution is too small and none too large.

**Kingston Victoria Hospital, Kingston-on-Thames,** commenced the year with a deficiency of £133, and this burden it has had to carry through the first half of 1918. It has entailed a more or less constant overdraft at the bank, and the tendency is for the deficiency in the account to increase rather than diminish as the year advances. Unless unexpected financial help is forthcoming the committee will be forced to make a special appeal for funds in the autumn, as it is determined the work of the hospital shall not be impaired.

**London Homœopathic Hospital, Great Ormond Street, W.C. 1.**—The annual report bears a striking testimony to the benefits conferred by the hospital and to the great success which attends the efforts of the management to get the utmost out of the funds which it has to administer. Since 1915 eighty-five beds have been placed at the disposal of the Admiralty for naval casualties, which are being treated by the civilian medical and surgical staff of the hospital, in addition to the ordinary civilian patients.

**London Hospital, Whitechapel, E.**—The work of the London Hospital, like that of all other voluntary hospitals, is carried on with great difficulty. Shortage of staff, both medical and lay, has necessitated considerable reorganisation in the hospital's work, although every endeavour is made to prevent unnecessary suffering and inconvenience to the sick poor. For the moment the hospital is not taking in wounded men, but has promised to do so as soon as the pressure on the War Office becomes acute. During the time that some of its beds were lent to the War Office nearly 4,000 wounded men were treated. Four floors are at present set aside for the use of wounded and sick officers, of whom 1,784 have so far been treated. This year is the hospital's quinquennial appeal year.

**London Lock Hospitals, Harrow Road, W.9, and Dean Street, W.1.**—The board have recently opened a new children's ward, in which there is not a single vacant bed. The history of the cases in this ward form most pathetic reading if the public could only know about them. It is very important that subscribers to charities should be aware that hygienist and moralist have worked side by side for over 170 years, and that the patients' minds are kept constantly occupied in between their treatment by means of classes, lectures, and instruction in domestic duties, sewing, etc., so that the hospital really fulfuls a dual purpose; and, further, every effort is made after the honorary surgeons have discharged the girls from the wards for situations to be found for them, or negotiations entered into to find them a suitable home or lodging to return to. £9,000 annually is required to maintain the charity.

**Metropolitan Ear, Nose, and Throat Hospital, Fitzroy Square, W.**—This special hospital has since the first days of the war been treating sick and wounded soldiers suffering from affections of the ear, nose, and throat, and now finds itself badly in need of funds. Notwithstanding the strain on its resources it has in its wards for women and children continuously carried on as usual, and through the depreciation in the value of money and the increase in cost of everything, notwithstanding the exercise of the strictest of economy, finds the outlook to be gloomy. Founded as far back as 1838, it is the oldest special hospital of its kind.

**Middlesex Hospital (The), W.**—The total income from all sources available for the maintenance of the general wards in 1917 was £38,148 19s. 1d., as compared with £45,644 6s. in the previous year. With a reduced

**HOSPITALS AND THEIR SPECIAL NEEDS—**
(*Continued*).

income and the added burden of increased prices the hospital's resources are now strained to the utmost. The care of the sick and suffering, in normal times a service worthy of support, is now a matter of supreme importance, when the health of the community ranks as a national asset which has to be realised. The Middlesex Hospital has done much to preserve that security. It is not generally known that, in addition to fulfilling all its obligations to the public as a general hospital, it maintains a special cancer charity.

**Mildmay Mission Hospital.**—The great aim and object is the fulfilment of our Lord's injunction, "Heal the sick . . . and say unto them, the Kingdom of God is come nigh unto you." Fifty doctors and twenty-four qualified nurses have gone from the hospital to work in the foreign field, and 213 women missionaries have received training in the out-patients' department for work abroad. Subscriptions and donations should be sent to the Hon. Treasurer, Mildmay Mission Hospital, Austin Street, E. 2.

**National Hospital for Diseases of the Heart, Westmoreland Street, W. 1.**—The number of in-patients treated during 1917 was 196, the small number being due to the length of treatment necessary in heart cases. The number of out-patient attendances was 17,116. Eighteen beds, which the hospital was unable to use for civil cases owing to want of funds, are now assigned to discharged disabled men sent by the Ministry of Pensions, and these are nearly always fully occupied. The hon. medical staff has examined and reported on upwards of 11,000 recruits with doubtful cardiac conditions. The financial position is very difficult, and the great need of the hospital is additional support.

**National Hospital for the Paralysed and Epileptic, Queen Square, W.C.1.**—The chief extra activities of this hospital at the present time are in the direction of extra accommodation for discharged and disabled sailors and soldiers. On behalf of the Ministry of Pensions the hospital is arranging to open an annexe in Queen Square for men suffering from both epilepsy and paralysis. A hospital at Clapham Park of thirty-two beds has been taken over for the treatment of severe cases of paralysis; and one at Maidenhead, with accommodation for fifty beds, will shortly be opened for neurasthenic and shell-shock men. The hospital continues its ordinary work without interruption, and at present there are in the wards seventy soldiers suffering from all forms of nerve injury and disease. Nearly 50,000 out-patients attended the hospital during 1917. In view of the great increase in the cost of maintenance, fresh donors and subscribers are urgently needed.

**Nelson Hospital for Wimbledon, Merton, and District.**—The expenses of maintenance are naturally in these times rising, so that subscriptions and donations are badly required to meet the increase. Last year the accounts showed a considerable deficit. A serious trouble has manifested itself in the heating apparatus, which will require an outlay of £300 or £400. This must be attended to for the safety of patients and resident staff.

**Paddington Green Children's Hospital, W. 2.**—Last year 742 patients were received into the hospital, 11,271 new out-patients were treated, and 41,356 out-patient attendances were recorded; 141 patients were received at the convalescent home at Slough. At the latter end of 1916 the hospital was in grave danger of being closed down owing to lack of funds. There is an annual deficiency of £3,500.

**Poplar Hospital for Accidents, East India Dock Road, E. 14.**—The hospital has never been in debt. The policy is to do the work with absolute efficiency within the means provided by friends, and it is fairly claimed that to-day the hospital is as perfect for its purpose as any hospital in London—in other words, though out of debt, not out of date. Please help the hospital to live up to its principles.

**Queen's Hospital for Children, Bethnal Green,** has increasing numbers of patients, and burdens growing through increased cost of supplies. The committee appeals to all friends of the children's cause. £10,000 extra income is badly needed before the end of the year.

**Queen Charlotte's Lying-in Hospital, Marylebone Road, N.W.**—Since the outbreak of war over 5,000 sailors' and soldiers' wives have either been admitted to the wards or attended and nursed in their own homes. A soldier, writing from the trenches, recently said: "I am writing to thank you for the great kindness and care that you have shown to my wife during the past week. I cannot express my gratitude sufficiently in words for the care and attention that you have given her in bringing her through the peril and bringing our dear little one into the world; it has been a great relief to my anxiety to know that she has been in good, kind hands. If only we in the line knew that our dear ones were always being looked after so well, our worry of home would be greatly lessened. God bless and prosper your humane work!"

**Royal Eye Hospital, Southwark, S.E.**—The Royal Eye Hospital, the only ophthalmic hospital in South London, set in the midst of a densely-populated working-class district. New out-patients last year, 19,198, including 3,270 casualties; out-patients' attendances, 56,097; in-patients admitted during the year, 550. Total ordinary income, £5,531; total expenditure, £5,963. Funds are urgently needed.

**Royal Free Hospital, Gray's Inn Road, W.C. 1.**—The adverse balance on account of maintenance for 1917, which it is feared during the current year will amount to several thousand pounds, is a matter which causes grave concern to all responsible for the hospital's welfare. There is, in addition, the responsibility for clearing off a debt of £21,000 to the hospital bankers. Funds are urgently required for current maintenance, as well as for the special purpose of providing improved accommodation for the nursing staff.

**Royal Hospital for Diseases of the Chest, City Road, E.C. 1.**—An urgent special appeal for £25,000 is being issued on behalf of this hospital, which for over a century has proved its usefulness by restoring health to thousands. The ordinary work of the hospital is now cut down to the out-patients; but, partly subsidised for the time by the War Office, the hospital is full of sick and wounded soldiers. Funds are most urgently needed.

**Royal London Ophthalmic Hospital, City Road, E.C.,** which is well known to the suffering poor as Moorfields Eye Hospital, earnestly appeals for help to

### HOSPITALS AND THEIR SPECIAL NEEDS—

*(Continued.)*

enable it to carry on its work. During the first three months of this year (January to March) 578 in-patients were admitted, and the daily average number of beds occupied was 116. The average number of out-patients' attendances daily for the same period was 328. Large numbers of munition workers are treated for foreign bodies in the eye.

**Royal National Hospital for Consumption, Ventnor.** By reason of the increased cost of all supplies the expenditure for the efficient maintenance of this hospital is now some £4,000 per annum in excess of pre-war times—a heavy responsibility upon the management. All the beds—160 in number—are constantly occupied, and many invalids from the Navy and Army are under the care of the medical staff.

**Royal Sea-Bathing Hospital, Margate.**—This is a unique hospital, being devoted to the treatment of the surgical forms of tuberculosis. The 200 beds are all occupied, and there is a waiting-list of fifty applicants, some of whom are ex sailors and soldiers. There is room for extension, but an increase in the number of patients would involve the provision of additional accommodation for nurses, servants, etc., which would necessitate a large expenditure for which funds are urgently needed. The office is at 13 Charing Cross, S.W. 1.

**Royal Waterloo Hospital for Children and Women, Waterloo Road, S.E. 1.**—There is an assured income of only £3,000 to meet an annual expenditure of £11,000. The institution has to rely on voluntary contributions during the year to make good this deficit of £8,000. The present loan at the bank is £2,000. The hospital is situated in one of the poorest parts of S.E. London; its work is confined to the children and women of the poor, two-thirds of the accommodation being reserved for children.

**Royal Westminster Ophthalmic Hospital, King William Street, Strand, W.C. 2.**—This hospital was established 100 years ago, and the demands upon its resources have been greatly increased, as the ophthalmic departments of many of the general hospitals have been either entirely closed or reduced. Accommodation has been provided for a large number of wounded soldiers, in addition to the requirements of the civilian population. In these circumstances the committee appeal for increased support.

**St. Andrew's Hospital, Dollis Hill, N.W. 2.**—For gentlepeople unwilling to go into the free wards of a public hospital and unable to meet the fees of nursing homes. Its work is undenominational. The building debt now stands at £6,717, and the deficit on the 1917 current account is £74.

**St. George's Hospital, S.W. 1.**—Despite the many difficulties arising out of the strenuous times in which people are at present living, the work of this hospital is being carried on as efficiently and with just as good results as before the war. In 1917, 4,402 in-patients and 29,605 out-patients received treatment. Of the former, 936 were sent to the convalescent branch at Wimbledon, at no cost to themselves. During the year 514 sick and wounded soldiers were admitted, which brings the total number treated at this hospital up to the end of 1917 to 1,689. As some indication of the severity of the cases admitted, it may be mentioned that during the year there were 1,626 major operations performed. The income account shows a deficit on the year's working of £6,947, and, as far as can be ascertained at present, there is likely to be a similar deficit this year.

**St. John's Hospital for Diseases of the Skin, Leicester Square, W.C. 2.**—This hospital is in its fifty-fifth year of work in the interests of those suffering from the peculiarly distressing and irritating diseases which affect the skin. Facial disfigurement in many cases results from these diseases when skilled advice is not sought in time. The children's ward is closed owing to lack of funds, and special help is invited to enable the board of management to reopen this ward. Donations are earnestly appealed for.

**St. Mark's Hospital for Cancer, etc., City Road, London, E.C. 1.**—This hospital wishes to buy an adjoining piece of land for extension when the proper time comes. The erection of a factory thereon, shutting out light and air from suffering patients, would be an injury too serious to contemplate, and the good work which has been carried on for over seventy years would be impeded. The Lord Mayor has issued an appeal for funds to purchase the site.

**St. Mary's Convalescent Home, Birchington-on-Sea.** Lady Fremantle makes an urgent appeal for help towards the funds of the home, where poor women and girls are received who need rest and refreshment, also mothers and babies.

**St. Mary's Hospital for Women and Children, Plaistow, E. 13.**—Last year was the first occasion for many years when the expenditure of this hospital exceeded its income. The deficit on maintenance account amounted to £588 15s. 9d., and on capital account (buildings) to £553 0s. 8d. The expenditure on capital account was necessitated by the urgent need of improvement in the nurses' and servants' quarters. At the outbreak of war a scheme for the rebuilding of the nurses' home, etc., and the out-patient department was postponed, but the prolongation of hostilities rendered it imperative that temporary improvements should be carried out, and, as soon as possible, the permanent buildings should be commenced.

**St. Monica's Home Hospital for Sick Children, 16 Brondesbury Park, N.W. 6.**—This hospital, which is primarily for cases requiring a long course of treatment, such as tubercular hip, spine, etc., has suffered considerably during the war, and at the end of 1917 there was an accumulated deficit of £638 on the income and expenditure account.

**St. Peter's Hospital, Covent Garden, W.C.**—Thousands of men have to thank St. Peter's Hospital for peace and comfort in their declining years, some through the relief obtained within its walls, others through its influence on surgical practice. The latter, possibly through not having heard its name, have been unable to express their gratitude by giving it their material support. A sum of £10,000 is needed to meet pressing liabilities.

**St. Thomas's Hospital.**—This hospital has done much good work during the past year; the civilian population have been cared for as usual, and the military section of the hospital has been fully occupied, many very serious cases having been sent here for treatment on arrival from the various seats of war. The governors have heartily adopted the scheme of the Ministry of Pensions for the efficient treatment of that sadly increasing and fully deserving class—the invalid war pensioners. The staff have loyally

*(Continued on p. x.)*

